# Molecular simulation of SARS-CoV-2 spike protein binding to pangolin ACE2 or human ACE2 natural variants reveals altered susceptibility to infection

**DOI:** 10.1099/jgv.0.001452

**Published:** 2020-06-15

**Authors:** Jingfang Wang, Xintian Xu, Xinbo Zhou, Ping Chen, Huiying Liang, Xuan Li, Wu Zhong, Pei Hao

**Affiliations:** ^1^​ Key Laboratory of Molecular Virology and Immunology, Institut Pasteur of Shanghai, Center for Biosafety Mega-Science, Chinese Academy of Sciences, Shanghai 200031, PR China; ^2^​ University of Chinese Academy of Sciences, Beijing 100039, PR China; ^3^​ National Engineering Research Center For the Emergence Drugs, Beijing Institute of Pharmacology and Toxicology, Beijing 100850, PR China; ^4^​ Key Laboratory of Synthetic Biology, CAS Center for Excellence in Molecular Plant Sciences, Chinese Academy of Sciences, Shanghai 200032, PR China; ^5^​ The Joint Program in Infection and Immunity, a. Guangzhou Women and Children's Medical Center, Guangzhou Medical University, Guangzhou, 510623, China; and b. Institute Pasteur of Shanghai, Chinese Academy of Sciences, Shanghai 200031, PR China

**Keywords:** SARS-CoV-2, coronavirus, ACE2 variants, pangolin, susceptibility, molecular dynamic simulation

## Abstract

We constructed complex models of SARS-CoV-2 spike protein binding to pangolin or human ACE2, the receptor for virus transmission, and estimated the binding free energy changes using molecular dynamics simulation. SARS-CoV-2 can bind to both pangolin and human ACE2, but has a significantly lower binding affinity for pangolin ACE2 due to the increased binding free energy (9.5 kcal mol^−1^). Human ACE2 is among the most polymorphous genes, for which we identified 317 missense single-nucleotide variations (SNVs) from the dbSNP database. Three SNVs, E329G (rs143936283), M82I (rs267606406) and K26R (rs4646116), had a significant reduction in binding free energy, which indicated higher binding affinity than wild-type ACE2 and greater susceptibility to SARS-CoV-2 infection for people with them. Three other SNVs, D355N (rs961360700), E37K (rs146676783) and I21T (rs1244687367), had a significant increase in binding free energy, which indicated lower binding affinity and reduced susceptibility to SARS-CoV-2 infection.

As of 13 May 2020, coronavirus disease 2019 (COVID-19) has caused close to 4 170 000 infections and more than 287 000 deaths worldwide [1]. We first reported that the novel coronavirus SARS-CoV-2 (2019-nCoV), the causative agent for the epidemic, is transmitted between humans via the spike protein–angiotensin-converting enzyme II (ACE2) interaction pathway, which is the mechanism through which 2003 SARS-CoV infects human cells [2]. Human ACE2 was previously found to be the functional receptor for SARS-CoV transmission between humans [3]. A recent published study showed that SARS-CoV-2 infected HeLa cells in an ACE2 expression-dependent manner [4]. The C-terminal RBD domain of the spike protein binds to human ACE2 through interactions with its N-terminal peptidase M2 domain [5]. In ACE2, 18 amino acid residues located in 4 regions around the N-terminal alpha-helical lobes were critical for interaction with the RBD domain of SARS-CoV spike protein [6].

Coronaviruses closely related to SARS-CoV-2 were identified in pangolins (*Manis javanica*), which were considered to be a candidate for an intermediate host [7]. Our analysis showed that pangolin-associated betacoronaviruses assembled from metagenomics sequences had ~90 % sequence identity to SARS-CoV-2, lower than the 96.2 % sequence identity between SARS-CoV-2 and the bat coronavirus RaTG13 detected in *Rhinolophus affinis* [4]. To address the question of whether pangolins might the intermediate host for SARS-CoV-2, we first examined whether SARS-CoV-2 spike protein can bind to pangolin ACE2, the orthologue of the human receptor for SARS-CoV-2. If it can, how strong is the interaction compared to that between SARS-CoV-2 spike protein and human ACE2?

We first constructed the complex models of SARS-CoV-2 spike protein binding to pangolin and human ACE2 based on sequence alignment (Fig. S1, available in the online version of this article), as described previously [8] (Fig. 1a, middle and left panels). The computational model of the human ACE2–spike complex was in excellent agreement with the recent crystal structures (PDB entry 6vw1) [9] and cryo-EM structures (PDB entry 6m17) [10]. The backbone root-mean-square deviation (RMSD) of the computational model for ACE2 and spike was only about 0.83 and 1.52 Å from the cryo-EM structure and 1.45 and 1.40 Å from the crystal structure (Fig. S2). To estimate the binding free energy for the complexes, we used molecular dynamics simulation (MDS) to refine the structure of the complexes.

**Fig. 1.**
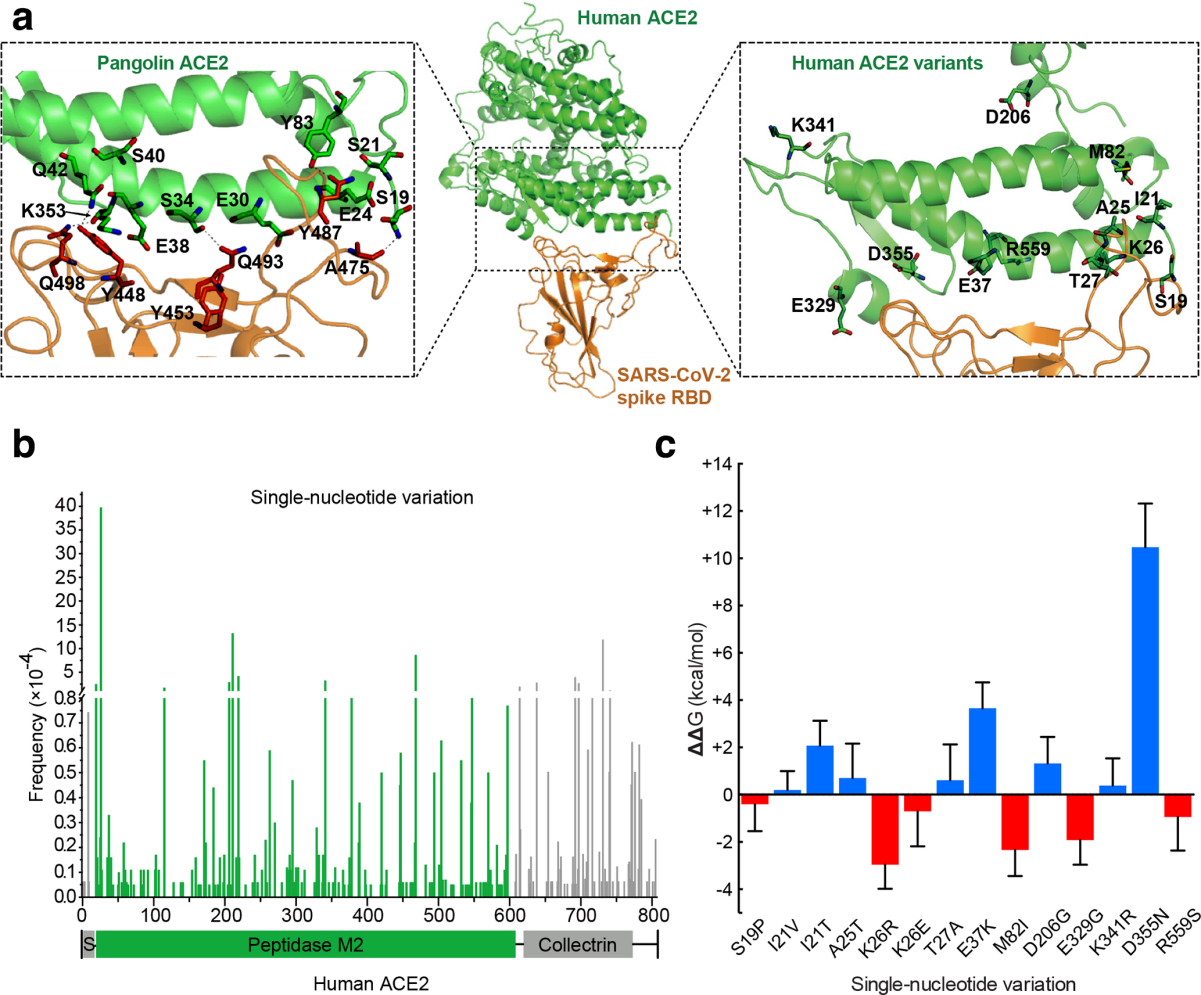
Structural modelling of pangolin ACE2 or human ACE2 variants binding to the SARS-CoV-2 spike RBD domain, and identification of missense human ACE2 genetic variants that induced changes in binding free energy for ACE2–spike interaction. (a) Middle panel: structural model of the binding complex between human ACE2 and the SARS-CoV-2 spike RBD domain, shown in a ribbon representation. Left panel: altered amino acid residues around the binding interface in pangolin ACE2. Right panel: human ACE2 variants around the binding interface. (b) Distribution of 317 missense single nucleotide variations (SNVs) in the human ACE2 gene and their overall allele frequency. The domain structure of ACE2 is presented below. S, signal peptide; green bars, SNVs located in the peptidase M2 domain. (c) Estimation of changes in binding free energy difference (ΔΔG) for interaction between the ACE2 variants and the SARS-CoV-2 spike protein. The stability of a binding complex depends on the difference in binding free energy (ΔG) between the binding configuration and the subunit-separating configuration. The ΔG of the wild-type ACE2–spike protein complex was used as the baseline and was subtracted from the ΔG of ACE2 variant–spike binding complexes. Red bars indicate a reduction in ΔG and hence stronger affinity for the SNVs binding to spike protein, while blue bars indicate the opposite. The ΔΔG values and their standard deviations were calculated using the metafor package in R 3.5. ΔKd was computed as the fold change of the dissociation constant between the SNVs and the wild-type. The ΔKds of these 14 missense SNVs are 1.92, 0.82, 0.03, 0.32, 122.54, 0.37, 0.00, 44.76, 0.12, 22.62, 0.68, 0.00 and 4.60, from left to right.

In brief, the initial structure of the complexes was treated using the LEaP module in the Amber 18 suite and parameterized using Amber ff14SB force field parameters [11]. After being neutralized and solvated in explicit TIP3P water surroundings, the complexes were subjected to energy minimization. MDS was performed for 1 ns with periodic boundary conditions and NPT ensemble (310 K and 1 bar) with the Amber 18 package [12]. The RMSD values for the backbone atoms of both ACE2 and spike proteins were calculated, which indicated that the simulation systems were equilibrated (Table S1). The binding free energy of the complexes was computed using molecular mechanics through the generalized Born surface area (MM-GB/SA) method [13]. The binding free energy for pangolin and human ACE2–spike binding complexes was averaged from 100 simulated configurations.

The binding free energy for human ACE2 and the SARS-CoV spike was −60.64±9.10 kcal mol^−1^, nearly 2.1 kcal mol^−1^ lower than that of human ACE2 and the SARS-CoV-2 spike (−58.55±8.75 kcal mol^−1^). As ∆G=RTlnKd, the binding free energy change of 2.1 kcal mol^−1^ corresponds to a nearly 30-fold dissociation constant, *K*_d_. The surface plasmon resonance results indicated that the binding affinity of ACE2 to the SARS-CoV-2 spike was 20-fold higher than that for ACE2 binding to the SARS-CoV spike. Thus, the computational ∆G is in good agreement with the experimental binding affinity [14].

As a result, compared to the human ACE2–spike binding complex, the pangolin ACE2–spike complex had a binding free energy increase of 9.5 kcal mol^−1^. This indicates that compared to human ACE2, pangolin ACE2 had a significantly lower binding affinity to the SARS-CoV-2 spike protein. In addition, the ΔΔG we calculated for the pangolin ACE2–spike complex is consistent with the results calculated independently by a different study [15]. Looking more closely into the contribution of different residues to the binding free energy increase in the pangolin ACE2–spike complex, the mutations E24 and E30 increased binding free energy by 2.14 and 1.12 kcal mol^−1^ (Fig. 1a, left panel). Although mutation S40 had very little contribution to binding free energy increase, it caused neighbouring Y41 and Q42 to both have increased binding free energy. These accurate results indicated that SARS-CoV-2 can bind to both pangolin and human ACE2, but has a significantly lower binding capacity for pangolin ACE2. These results would indicate that pangolins are less susceptible to SARS-CoV-2 than the human host. If pangolins were the intermediate host, this might suggest that SARS-CoV-2 may have adapted to the human host during animal-to-human transmission.

While the COVID-19 epidemic was spreading globally, there was an indication of likely differences in viral transmissibility, vulnerability and clinical severity for SARS-CoV-2 in different countries or regions. For example, very few locally transmitted COVID-19 cases have surfaced across African region to date, whereas the number of confirmed cases rose quickly in other regions [1]. Genetic variation is one of the important factors affecting the spread and severity of epidemics. In addition to genetic variation, people’s social behaviour, virus strain and mutation rate also have significant impacts on viral transmission rates. While those involved in SARS-CoV-2 transmission, clinical symptoms and prognosis have been poorly understood, the genetic variants of ACE2, the human receptor for SARS-CoV-2, stood out as probably the most influential genetic factor for vulnerability to SARS-CoV-2 and clinical progress of COVID-19. The natural genetic variants of ACE2 may alter human susceptibility or response to SARS-CoV-2 infection, which is an important question to address in this the health emergency.

ACE2 is among the most polymorphous genes in human populations, with over 10 000 different variants of human ACE2 having been recorded at dbSNP [16]. Early genetic studies identified some ACE2 variants that were associated with hypertension or chronic kidney disease in humans [17]. After initial analysis of all human ACE2 polymorphisms from dbSNP, we identified 317 SNVs that induced amino acid changes (missense mutation) in ACE2 (Fig. 1b and Table S2). Among these, 218 SNVs were located in the peptidase M2 domain of human ACE2 that may affect binding to the SARS-CoV-2 spike protein.

To identify the ACE2 genetic variants that affect spike protein binding, we first scanned the interface of the human ACE2–spike binding complex. Fourteen missense SNVs that likely affect ACE2 and spike interaction were selected for further analysis (Fig. 1a right panel, and Table S3). Thirteen of the 14 SNVs, with the exception being rs1016777825 (R559S), fell in the 4 ACE2 regions that were critical for interaction with the RBD domain of spike protein [6]. The wild-type ACE2–spike binding model was reconfigured with the substitution of each of the 14 SNVs. The binding free energy for each substitution was estimated using the newly refined structure as described above, and was compared to that of the wild-type ACE2–spike binding complex. Three of the missense SNVs, M82I (rs267606406), E329G (rs143936283) and K26R (rs4646116), showed a significant decrease in binding free energy (*t*-test, *P* value <0.05, Table S3), which would indicate higher binding affinities to spike protein, and thus greater susceptibility to SARS-CoV-2 infection (Fig. 1c). In the wild-type ACE2, the Lys26 had an unfavourable contribution (0.51 kcal mol^−1^) to the binding free energy. Its substitution with arginine at this position reduced the unfavourable contribution (0.32 kcal mol^−1^), and also reduced the contribution of Asp30 and His34 to the binding free energy (−0.55 and −0.24 kcal mol^−1^, respectively). Notably, the K26R mutation strengthened the hydrogen bond between His34 and Tyr453 of the SARS-CoV-2 spike. Similarly, M82I and E329G mutations had an impact on Tyr83 and Gln325, enhancing the hydrogen bonds between Tyr83 and Tyr489 of the spike protein, and between Gln325 and Gln506 of the spike. Based on GnomAD exomes data [16], SNV rs4646116 (K26R) had a population allele frequency (AF) of 0.012 in Ashkenazi Jewish, 0.005 in European, 0.0033 in American and 0.0008 in Asian populations (Table S3). SNV rs4646116, which exhibited significantly higher binding affinity to the SARS-CoV-2 spike protein, will increase susceptibility to SARS-CoV-2 infection for people who have it.

On the other hand, three other SNVs, D355N (rs961360700), E37K (rs146676783) and I21T (rs1244687367), showed a significant increase in their binding free energy (*t*-test, *P* value <0.05, Table S3), which indicates lower binding affinity to spike protein, and reduced susceptibility to SARS-CoV-2 infection. Both E37K and D355N mutations increased their contribution to binding free energy by 3.32 and 4.52 kcal mol^−1^, respectively. These increases were caused by changes in surface charge. The substitution of Glu37 by lysine reduced the surface charges, increasing the electrostatic contribution to the binding free energy from −0.06 to 3.26 kcal mol^−1^. Similarly, D355N mutation increased the electrostatic contribution to the binding free energy from −7.02 (wild-type) to −2.50 kcal mol^−1^, an increase of ~4.52 kcal mol^−1^. Based on GnomAD exomes data, SNV rs146676783 (E37K) had an AF of 0.0001 in both European and African populations. It would reduce susceptibility to SARS-CoV-2 infection for people who have it.

Additionally, we searched in the 2019 Novel Coronavirus Resource at the National Genomics Data Center (NGDC) (https://bigd.big.ac.cn/ncov/variation/annotation) for mutant SARS-CoV-2 strains. We found that to date SARS-CoV-2 mutants have mostly occurred in the orf1ab gene, with very few in the spike gene. Although the chance of them directly affecting interaction with the ACE2 variant is small, it would be interesting to model how the spike mutant residues affect spike interaction with human ACE2 and ACE2 variants when more spike mutation data have been accumulated.

In summary, we constructed the complex models of SARS-CoV-2 spike protein binding to pangolin or human ACE2, and estimated the binding free energy changes using MDS. SARS-CoV-2 can bind to both pangolin and human ACE2, but has a significantly lower binding affinity for pangolin ACE2, resulting from the increased binding free energy of 9.5 kcal mol^−1^ for pangolin ACE2 compared to human ACE2. ACE2 is among the most polymorphous genes in human populations, for which we identified 317 missense SNVs from dbSNP database. Several missense ACE2 SNVs were found to significantly alter binding capacity to the SARS-CoV-2 spike compared to wild-type ACE2. Three SNVs, E329G (rs143936283), M82I (rs267606406) and K26R (rs4646116), had a significant reduction in binding free energy, which indicates higher binding affinity than wild-type ACE2 and greater susceptibility to SARS-CoV-2 infection. Three other SNVs, D355N (rs961360700), E37K (rs146676783) and I21T (rs1244687367), had a significant increase in binding free energy, indicating lower binding affinity and reduced susceptibility to infection. These findings via molecular simulation provide a framework for future studies to experimentally validate the susceptibility of ACE2 natural variants to SARS-CoV-2 infection.

## Supplementary Data

Supplementary material 1

Supplementary material 2

Supplementary material 3

Supplementary material 4
