# Supplementary material for: Molecular simulation of SARS-CoV-2 spike protein binding to pangolin ACE2 or human ACE2 natural variants reveals altered susceptibility to infection
Source: J Gen Virol. 2020 Jun 15;101(9):921–4. doi: 10.1099/jgv.0.001452 (PMC7654750; doi:10.1099/jgv.0.001452)
Supplement: Supplementary material 1 [file jgv-101-921-s001.pdf]

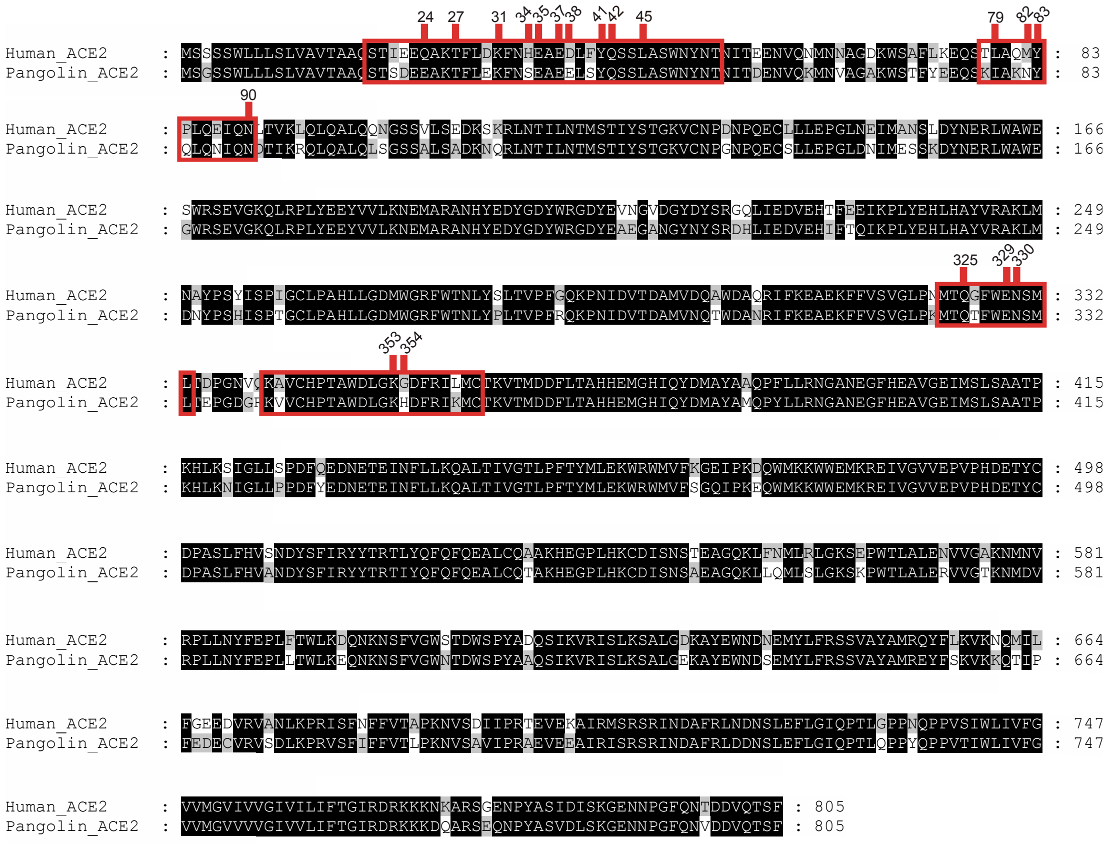


**Figure S1** Sequence alignment of pangolin and human ACE2 protein sequences. Residues 24, 27, 31, 34, 35, 37, 38, 41, 42, 45, 79, 82, 83, 90, 325, 329, 330, 353 and 354 (numbered based on human ACE2 sequence) are critical residues for interaction with RBD domain of SARS-CoV-2 spike protein. The NCBI accession ID for human and pangolin ACE2 is NP_068576.1 and XP_017505752.1, respectively.

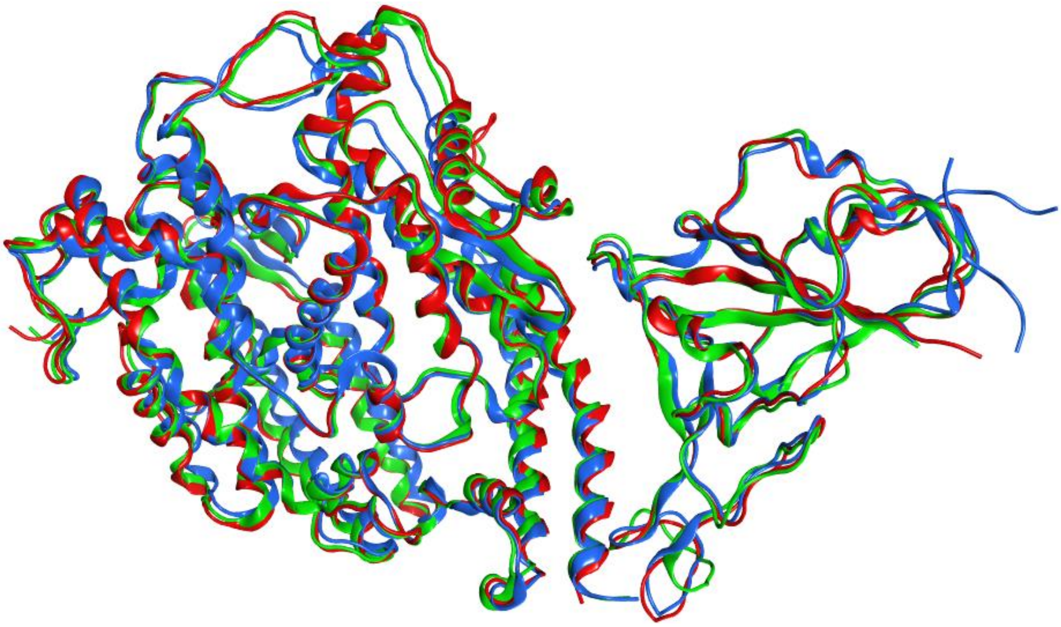

**Figure S2.** Structural comparison of the computational model with the crystal and cryo-EM structures. The backbone structures of the computational model, crystal structure (PDB entry 6vw1), and cryo-EM structure (PDB entry 6m17) were shown in ribbon representations and colored by green, blue, and red. The ACE2 backbone RMSD values for the computational model were 0.83 Å and 1.45 Å from the cryo-EM and crystal structures. The spike backbone RMSD values for the computational model were 1.52 Å and 1.45 Å from the cryo-EM and crystal structures.
